# Measurements of bone mineral density and stiffness index in young Saudi females

**DOI:** 10.12669/pjms.322.9757

**Published:** 2016

**Authors:** Lina Fahmi Hammad

**Affiliations:** Dr. Lina Fahmi Hammad, Department of Radiological Science, College of Applied Medical Sciences, King Saud University, Riyadh, Saudi Arabia

**Keywords:** QUS, DXA, SI, BMD, T-score, Saudi Female

## Abstract

**Objective:**

To examine the ability to use Quantitative Ultrasonography (QUS) densitometer for screening of osteoporosis and osteopenia by comparing QUS values obtained at the calcaneus region to bone mineral density (BMD) values measured at the spine and the neck of the femur using Dual Energy X-ray Absorbemetry (DXA).

**Methods:**

QUS (in the calcaneus region) and DXA (the spine and the neck of femur respectively) measurements were performed in 101 females.

**Results:**

The precision of the QUS parameters varied from 1.77-1.78, whereas the reliability ranged from 92.2%-98.6%. For the QUS parameters variability between subjects was greater than that within subjects. Positive correlation were found between stiffness index (SI) and BMD_spine_ and BMD_N.femur_ (r= 0.29 & r=0.25 respectively, P < 0.05) and a strong positive correlation between T-score_calcaneus_ and both T-score_Spine_ and T-score_N.femur_ (r= 0.5 & r=0.58 respectively, P < 0.01).

**Conclusions:**

QUS is a reliable technique to be used in combination with DXA for the investigation of osteopenia and osteoporosis.

## INTRODUCTION

Bone is a dynamic organ which provides structural support for the body, protection of vital organs, storage of crucial nutrients, minerals, and lipids and production of blood cells. It is made of a relatively hard composite material that is constantly remodeling to adapt to the daily forces placed upon it, when an imbalance between bone resorption and bone formation happens, osteoporosis occurs. QUS was proposed as an indirect assessment of bone quality, as the modality has the advantages of being portable, with no ionizing radiation used, in addition to comparable cost and time effectiveness in relation to the gold standards for measuring bone density; DXA and Quantitative computed tomography (QCT).^1^,^2^

As DXA and QCT provide limited information on bone structure, the QUS stiffness index were suggested to assess dimensional structure and strength.^3^ The possible clinical applications of QUS include diagnosis of osteoporosis, monitoring of skeletal changes associated with disease prognosis and treatment, and fracture risk assessment.^4^ Previous studies within the region examined BMD and QUS separately, no studies where done to examine the ability of QUS to evaluate bone density in the young Saudi population.^5^-^7^

The aim of this study was to examine whether QUS stiffness index measured at the calcaneous area that can reflect the status of bone at the area most likely to endure fractures (spine and neck of femur), or whether a combined assessment of QUS and BMD should be used to assess bone loss at different areas.

## METHODS

### Study Design

One hundred and one young adult Saudi females attending King Saud University were enrolled in the study (age range 20–24.9 years). The exclusion criteria consisted of pregnancy, diseases or medication affecting bones metabolism, a previous history of fracture, and any terminal illness. Informed consent was obtained from all subjects, and the study was approved by local research ethics committee (CAMS 18/3536). Age, body weight (kg) and height (m) were recorded, body mass index (BMI) (kg/m^2^) was calculated. Both QUS in the calcaneus area and BMD in the lumbar spine at the area of L2-L4 (Spine) and femoral neck area (N.femur) were obtained for each subject.

### QUS measurements

A water-bath ultrasound system which generates a band of frequencies from 200 to 600 kHz (Lunar Achilles Insight™ - GE Healthcare) was used for measurements of SI in the calcaneoual region of the independent foot. Both SI (automatically calculated from broadband ultrasound attenuation and the speed of sound) and T-score were recorded using a standard protocol supplied by the manufacturer. Data collection was obtained after quality control was carried out using the quality phantom. Precision and reliability of the technique were examined by repeating the measurements on 20 subjects in three consecutive days.

### The BMD measurements

DXA scans (Lunar iDXA™ - GE Healthcare) were utilized to measure BMD (g/m^2^) in the lumbar spine (L2-L4) and the femoral neck area. A standard protocol supplied by the manufacturer was used, which included a quality control test using a standard phantom.

### Statistical Analysis

The variability within (MSw) and between (MSb) subjects for SI were examined using analysis of variance test (Anova test), precision (expressed as the coefficient of variation), and reliability (expressed as the intraclass correlation coefficient of reliability) for the SI were calculated using Minitab™ software. Using SPSS™ software, Pearson Correlation Coefficient was used to examine the presence of an association between SI, T-score values in the calcaneus and the BMD and T-score values in the lumber spine and the femoral neck area.

## RESULTS

Variability between subjects was greater than that within subjects, the measurements of precision and reliability of the Achilles densitometer parameters are tabulated in Table-I. The precision varied from 1.77-1.78, whereas the reliability ranged from 92.2%-98.6%. Age, anthropometric data, QUS and BMD values in the spine and neck of the femur are presented in Table-II.

**Table-I:** Reliability and precision of the three parameters measured by QUS the calcaneus area.

Parameter	Variability within subjects (MSw)	Variability between subjects (Msb)	Significance	Coefficient of variation %CV	Reliability R%	Precision
SI	5.050	1047.102	P<0.001	2.176%	98.6	1.7743
T- score	0.071	4.203	P<0.001	-1.396%	95.1	1.7779

**Table-II:** Descriptive measures mean and (SD) for age, height, weight, BMI, QUS parameter in the calcaneus area and DXA parameters for both the spine and the neck of the femur (No of subjects =101).

Age (Yrs.)	Height (m)	Weight (kg)	BMI	SI_Calcaneus_	T-score_calcaneus_	BMD_Spine_	T-score_Spine_	BMD_N.femur_	T-score_N.femur_
21.35 (0.83)	1.59 (0.07)	56.04 (9.47)	22.27 (3.65)	93.22 (19.4)	-0.48 (1.11)	1.1 (0.2)	- 0.77 (1.06)	0.97 (0.18)	-0.65 (0.88)

The association between QUS parameters (stiffness index and T-score calcaneus) and DXA parameters (BMD_spine_, T-score_spine_, BMD_N.femur_ and T-score_N.femur_) were investigated and tabulated in Table-III. Positive correlation were found between stiffness index and BMD_spine_ and BMD_N.femur_ (r= 0.29 & r=0.25 respectively, P < 0.05) and a strong positive correlation between T-score_calcaneus_ and both T-score_Spine_ and T-score_N.femur_ (r= 0.5 & r=0.58 respectively, P < 0.01).

**Table-III:** Pearson correlation coefficient of QUS and DXA parameters.

	SI	T-score_calcaneus_	BMD_Spine_	T-score_Spine_	BMD_N.femur_	T-score_N.femur_
T-score_Calcaneus_	0.79**					
BMD_Spine_	0.29**	0.29**				
T-score_Spine_	0.49**	0.5**	0.78**			
BMD_N,femur_	0.25*	0.32**	0.47**	0.55**		
T-score_N.femur_	0.5**	0.58**	0.63**	0.77**	0.63**	

* Correlation is significant at the 0.05 level.

** Correlation is significant at the 0.01 level.

## DISCUSSION

The presence of portable and cost effective measuring tools for the diagnosis of osteoporosis is essential; QUS presents itself as a non-destructive, non-ionizing, inexpensive technique for the assessment of the mechanical properties of bone, which would contribute to the detection of osteoporosis, and would also allow the formulation of a fracture threshold.^8^

SI, a parameter derived from speed of sound (SOS) and broadband ultrasound attenuation (BUA), was suggested as a variable to discriminate women with low bone density from healthy postmenopausal controls.^9^ The ability to use QUS as a screening tool to determine those who might be most beneficial from preventive measures will be of benefit.^10^

The presence of a relationship between stiffness index in the calcaneus and BMD in the spine and N._femur_ has not been examined, previous studies were conducted to establish reference values for SI and BMD values within the region in separate studies.^5^-^7^,^11^ Whether QUS results alone or a combined assessment of QUS and bone density can be used to predict future fracture risk in females is not clear, further studies are needed to determine if such possibilities can be achieved.

The aim of the study was to measure the precision and reliability of the QUS densitometer, to examine whether QUS parameters could reflect the status of bone at the area most likely to endure fractures,^12^,^13^ and to investigate the ability to use the technique for screening of osteoporosis and osteopenia.

High precision (≈ 1.77) and reliability (from 95% to 98%) were found in the two parameters measured by QUS, demonstrated by the small coefficient of variation and the variability between subjects being greater than within subjects, which mean that QUS enabled the measurements of significant differences between subjects for both SI and T-score (Table-I). Such findings are in agreements with previous studies,^14^ thus suggest the stability of the equipment and the ability of the technique to be used for follow up procedures when examining patients.

Mean values of SI in our study demonstrate similar values when compared to measurements obtained previously in the Eastern region of Saudi Arabia, Lebanon, China and Britain, but lower than that of American young females (Table-II).^11^,^14^-^16^ The SI obtained in this study was only compared with SI values mentioned in previous studies that have used similar devices due to the unavailability of cross-calibration method between various QUS devices present in the market.

Mean BMD values in this study exhibited lower values compared to data obtained from similar age group females from Kuwait, Qatar and Morrocco (Table-II).^17^-^19^ Although ethnic differences which affect bone density is ruled out when data from the Saudi community is compared to data obtained from other countries of the gulf, the reduction in BMD values in the Saudi females could be attributed to many factors including differences in the subjects selection protocol, possible inadequate accrual of bone during childhood and adolescence, lack of exercise and sedentary lifestyle with few outdoor activities, which may result in the possibility of an increase in the incidence of osteoporosis later in life. Such findings invite more research on the endogenic and exogenic factors affecting peak bone mass in the Saudi population.

A positive correlation was found between both QUS and DXA parameters with BMI attributed to the effect of weight only (results published previously),^6^,^7^ Similar results were reported previously, as increasing weight is known to be associated with higher bone density.^20^ Such findings in the Saudi population suggest the importance of considering body weight in the evaluation of patients in relation to the diagnosis of osteoporosis.^21^

The ability to examine the presence of a relationship between stiffness index in the calcaneus and BMD in the spine and femur is needed. Nevertheless, when QUS and BMD were measured along a single direction and at the same previous location, results were highly correlated in both in-vitro and in-vivo studies.^22^,^23^ Such correlation is not known in the literature, where measurements are obtained from different sites, as previous studies conducted to establish reference values for SI and BMD values were done separately.^5^-^7^,^11^ The presence of a positive relationship between SI in the calcaneus area and BMD both in the spine and femur region suggests that the mechanisms that effect bone density affects as well bone structure. This mechanism seems to vary at different body site hence the association being positive but not relatively strong (r = 0.29 & 0.25 respectively). These findings could be attributed to differences in bone structure at different sites in addition to other factors.^24^ Previous in-vitro studies found that ultrasound properties are dependent on the microstructure of the bone and the trabecular orientation^25^ with the QUS being affected by the microstructure of the bone more than bone mass,^4^ which resulted in the QUS parameter unlike BMD exhibiting anisotropic effect dependent on the direction of propagation.^23^

### Limitations of this study

Include the inability to extrapolate our results to other ultrasound and DXA machines, middle-aged, pre and post-menopausal females were not included in the study, these areas should be investigated in the future. In conclusion this study has found that the QUS is a reliable technique to be used with DXA for the investigation of osteopenia and osteoporosis.

## CONCLUSIONS

Nevertheless, results demonstrate that the association between T-score in the calcaneus and T-score in the spine and femur are stronger (r=0.57 & 0.59 respectively). Such findings suggest that during screening, it is possible to use T-score data obtained from QUS for referral to BMD measurements test, although QUS parameters on its own should not be used as a screening tool, or for the assessment of fracture risk.
